# A Chemotherapy Progression Decision Score for Treatment Selection After Initial Assessment in Pancreatic Ductal Adenocarcinoma

**DOI:** 10.1002/mco2.70903

**Published:** 2026-08-06

**Authors:** Yixin Zhang, Zhongquan Sun, Hongfan Ding, Changlin Zou, Linping Dong, Qi Xu, Jiali Ji, Xin Han, Hanqi Yu, Daqian Xu, Gaopeng Li, Yuhao Wang, Haoliang He, Yufeng Zhong, Xiaochang Wu, Jieer Ying, Weilin Wang, Yuan Ding

**Affiliations:** ^1^ Department of Hepatobiliary and Pancreatic Surgery The Second Affiliated Hospital, Zhejiang University School of Medicine Hangzhou Zhejiang China; ^2^ Key Laboratory of Precision Diagnosis and Treatment for Hepatobiliary and Pancreatic Tumor of Zhejiang Province Hangzhou Zhejiang China; ^3^ Research Center of Diagnosis and Treatment Technology For Hepatocellular Carcinoma of Zhejiang Province Hangzhou Zhejiang China; ^4^ Center For Medical Research and Innovation in Digestive System Tumors, Ministry of Education Hangzhou Zhejiang China; ^5^ Cancer Center Zhejiang University Hangzhou Zhejiang China; ^6^ ZJU‐Pujian Research & Development Center of Medical Artificial Intelligence for Hepatobiliary and Pancreatic Disease Hangzhou Zhejiang China; ^7^ Department of Radiotherapy and Chemotherapy The First Affiliated Hospital of Wenzhou Medical University Wenzhou Zhejiang China; ^8^ Department of Hepatobiliary and Pancreatic Surgery The Fourth Affiliated Hospital, Zhejiang University School of Medicine Yiwu Zhejiang China; ^9^ Department of Hepato‐Pancreato‐Biliary & Gastric Medical Oncology Zhejiang Cancer Hospital, Hangzhou Institute of Medicine (HIM), Chinese Academy of Sciences Hangzhou Zhejiang China; ^10^ Zhejiang Provincial Key Laboratory of Pancreatic Disease The First Affiliated Hospital, Institute of Translational Medicine, Zhejiang University School of Medicine, Zhejiang University Hangzhou China; ^11^ Department of Colorectal Surgery and Oncology of the Second Affiliated Hospital and Institute of Translational Medicine, Zhejiang University School of Medicine Hangzhou China; ^12^ Department of Hepatobiliary Surgery Huzhou Central Hospital, Zhejiang University Huzhou Hospital Huzhou Zhejiang China

**Keywords:** chemotherapy response, pancreatic ductal adenocarcinoma, prognostic prediction model, radiographic pseudoprogression, treatment decision‐making

## Abstract

Pancreatic ductal adenocarcinoma (PDAC) remains one of the most lethal malignancies, with chemotherapy response evaluation currently relying on the Response Evaluation Criteria in Solid Tumors (RECIST). However, RECIST inadequately captures the unique biology of PDAC, particularly stromal fibrosis–induced radiographic pseudoprogression. We developed and externally validated a multivariable prognostic prediction model, the chemotherapy Progression Decision (cPD) score, integrating biological markers and imaging metrics to guide therapeutic decision‐making after the first RECIST assessment. This multicenter retrospective study enrolled 616 PDAC patients across three cohorts: the Training Cohort (*n* = 155), Validation Cohort 1 (*n* = 263), and Validation Cohort 2 (*n* = 198). Multivariable Cox regression identified four independent prognostic predictors: neutrophil‐to‐lymphocyte ratio, baseline carbohydrate antigen 19‐9, tumor maximum cross‐sectional rate change ratio, and emergence of new lesions. These were integrated into an integer‐based score (8–13 points) stratifying patients into good prognosis (GP), poor prognosis (PP), and critical prognosis (CP) groups. The cPD model demonstrated robust discrimination and calibration, with significant survival differences across groups in all cohorts. Notably, the cPD score identified a subset of patients (GP and PP groups) for whom continuing the original chemotherapy yielded significantly better survival than switching regimens, even when RECIST classified the disease as progressive.

## Graphical abstract

Conventional RECIST criteria inadequately guide chemotherapy decisions in pancreatic ductal adenocarcinoma due to radiographic pseudoprogression. We developed and externally validated the chemotherapy Progression Decision (cPD) score in 616 patients across three cohorts. By integrating four readily available biological and imaging variables, the cPD score identifies patients who benefit from continuing original chemotherapy despite RECIST‐defined progression, enabling more refined therapeutic decisions

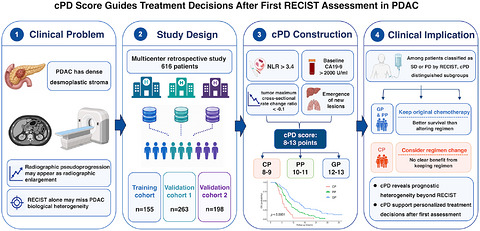

## 1 Introduction

Pancreatic ductal adenocarcinoma (PDAC) represents one of the most lethal malignancies worldwide, characterized by a 5‐year overall survival (OS) rate of only 13% and a mortality rate nearly equivalent to its incidence rate [1]. PDAC constitutes more than 90% of all pancreatic cancer cases and remains a formidable therapeutic challenge due to its late diagnosis, aggressive biology, and resistance to conventional therapies [2]. While radical surgical resection offers the only potentially curative approach, approximately 80% of patients present with locally advanced or metastatic disease at diagnosis, precluding upfront surgery and rendering systemic chemotherapy the cornerstone of management [3, 4]. Given the limited therapeutic options and the narrow window for clinical intervention in advanced PDAC, an accurate and standardized framework for evaluating chemotherapy response is essential for guiding subsequent treatment decisions—whether to continue the original regimen or to modify therapy—and ultimately improving patient outcomes.

The current standard for evaluating chemotherapy response in solid tumors is the Response Evaluation Criteria in Solid Tumors (RECIST 1.1), which primarily relies on unidimensional tumor size measurements [5]. However, RECIST has notable limitations when applied to PDAC. Unlike most solid tumors, PDAC is characterized by an exceptionally dense desmoplastic stroma that may comprise up to 80% of the tumor mass [6]. During chemotherapy, this stromal compartment frequently undergoes reactive fibrosis, lymphocytic infiltration, and inflammatory edema [7]. As a result, some PDAC patients may exhibit atypical treatment responses, such as *radiographic pseudoprogression*—defined as apparent radiographic tumor enlargement that does not reflect true disease progression—which substantially increases the complexity of efficacy assessment [8, 9]. Such cases may be erroneously classified as progressive disease (PD) under conventional RECIST criteria, leading to premature treatment discontinuation or unnecessary regimen alteration. Conversely, RECIST may also overlook biologically aggressive tumors that meet only stable disease (SD) criteria. These limitations underscore the inadequacy of imaging‐based unidimensional assessment alone and highlight the urgent need for a more comprehensive evaluation framework that integrates multiple biological and radiographic dimensions of treatment response.

Beyond imaging, several biological and laboratory markers have been investigated for prognostic assessment in PDAC. The neutrophil‐to‐lymphocyte ratio (NLR), an accessible inflammatory biomarker, has been associated with poor OS across multiple solid tumors, including PDAC [10, 11]. Carbohydrate antigen 19‐9 (CA19‐9), a tumor‐associated glycoprotein, remains the most widely used serum biomarker, with elevated baseline levels predicting worse outcomes [12, 13]. Nevertheless, each of these single markers has inherent limitations: NLR fluctuates with infection and concomitant medications [14]; CA19‐9 is uninformative in Lewis antigen–negative individuals, who comprise approximately 5%–10% of the general population and are constitutively unable to secrete this glycoprotein [15, 16]; and imaging metrics alone fail to capture the underlying biological tumor activity. Consequently, no single biomarker or imaging parameter has demonstrated sufficient accuracy to guide chemotherapy decision‐making in PDAC, and the standalone application of any individual indicator is unlikely to overcome the multidimensional complexity of treatment response in this disease.

Emerging evidence suggests that integrating multiple clinical, biological, and radiographic factors into a unified prognostic framework may provide superior predictive performance compared with any single indicator alone. Several prognostic scoring systems have been developed for advanced solid tumors, including the modified Glasgow Prognostic Score (mGPS) based on C‐reactive protein and albumin [17, 18], early tumor shrinkage (ETS) as an imaging‐based predictor of survival in PDAC patients treated with FOLFIRINOX [19, 20], and a recent simple scoring system incorporating tumor shrinkage rate, tumor density, and post‐chemotherapy CA19‐9 levels in resected locally advanced PDAC [21]. However, most existing models for PDAC have focused on baseline characteristics at diagnosis or post‐resection settings rather than on response assessment after chemotherapy initiation in unresectable disease, and few have incorporated dynamic imaging changes alongside biological markers in a clinically accessible scoring framework. Moreover, the absence of standardized protocols integrating longitudinal radiographic evaluation, molecular biomarkers, and patient clinical dynamics has impeded precision in therapeutic decision‐making after the first RECIST assessment.

In this study, we developed and externally validated a multivariable prognostic prediction model—the Chemotherapy Progression Decision (cPD) score—as a clinically accessible tool to guide treatment decisions for PDAC patients after the first RECIST assessment. Built upon a combination of biological markers (NLR, baseline CA19‐9), imaging metrics (tumor maximum cross‐sectional rate change ratio), and the emergence of new lesions, the cPD score integrates four independent prognostic predictors into an integer‐based scoring system, stratifying patients into three risk groups (Good Prognosis [GP], Poor Prognosis [PP], Critical Prognosis [CP]). The model was developed in a single‐center Training Cohort and externally validated in two independent multicenter cohorts. Beyond demonstrating model performance, the present work aims to clarify whether cPD‐guided treatment decisions (continuation vs. alteration of the original chemotherapy regimen) provide survival benefit beyond conventional RECIST‐defined classifications, thereby offering oncologists a practical bedside framework for personalized therapeutic decision‐making in advanced PDAC.

## 2 Results

### 2.1 The Three Cohorts Showed Generally Consistent Demographic and Tumor Characteristics

**FIGURE 1:**
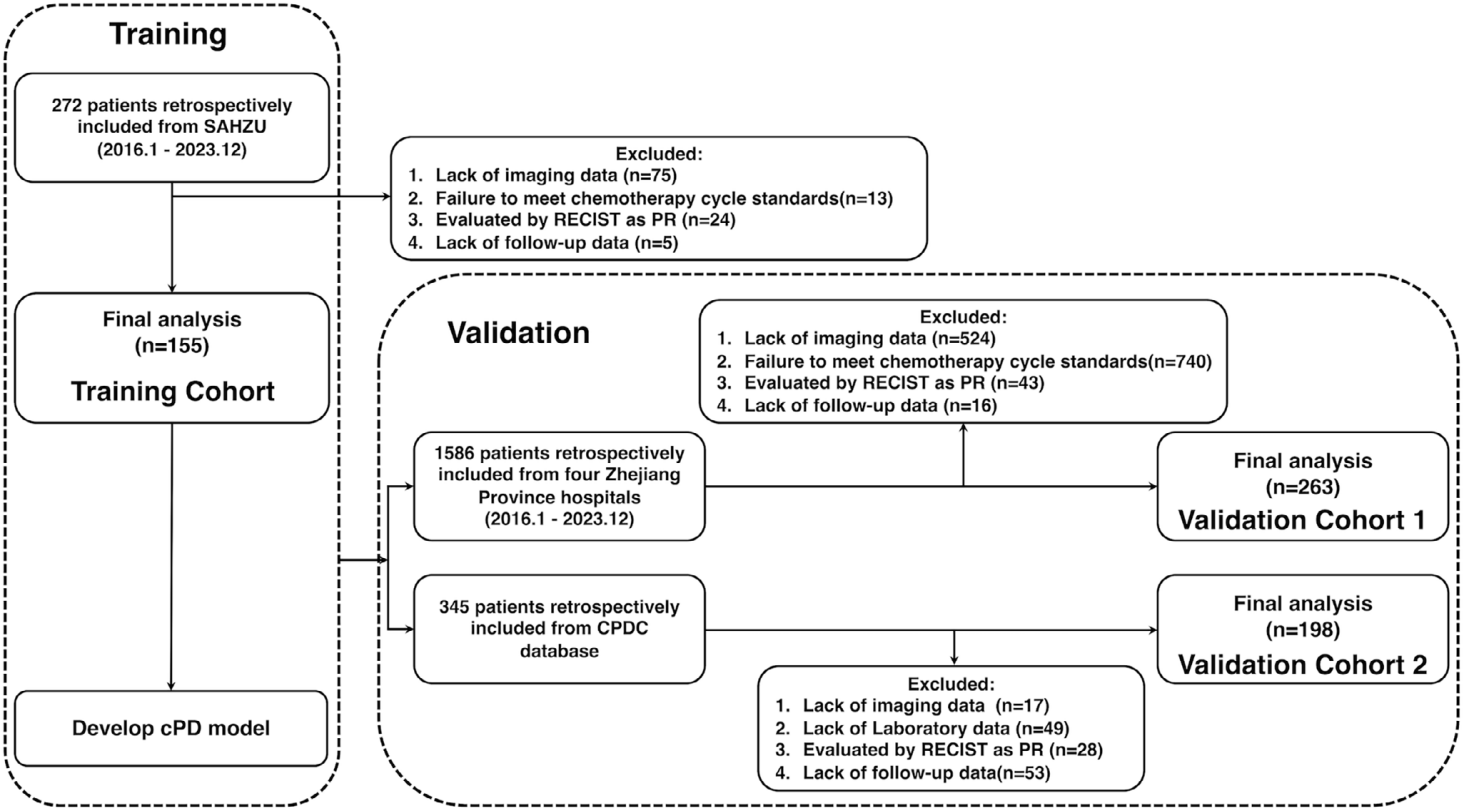
Patient flow diagram for the three cohorts.

To establish a comparable baseline for model development and validation, we systematically extracted and summarized the demographic, clinical, and laboratory characteristics of all enrolled patients across the three cohorts. Categorical variables were presented as counts and percentages, and continuous variables as medians with interquartile ranges (IQR). Of the 2203 patients initially screened, 616 met the inclusion criteria and were enrolled: Training Cohort (*n* = 155, the Second Affiliated Hospital of Zhejiang University [SAHZU]), Validation Cohort 1 (*n* = 263, four independent Zhejiang Province institutions), and Validation Cohort 2 (*n* = 198, the China Pancreas Data Center [CPDC]). The detailed flow diagram is presented in Figure 1. Among Training Cohort patients, 67% were male and the median age at diagnosis was 65 years (IQR: 58–70). Regarding initial resectability status determined by comprehensive radiographic imaging, 92 patients (59.35%) had combined distant metastases and 48 (30.97%) had locally advanced disease. As shown in Table 1, baseline demographics and tumor characteristics were comparable across the three cohorts, with no clinically meaningful differences in sex distribution, age, tumor location, or eighth edition American Joint Committee on Cancer (AJCC) stage.

**TABLE 1:** Demographic and clinical characteristics of patients.

Characteristic	Training Cohort	Validation Cohort 1	Validation Cohort 2
	(*n* = 155)	(*n* = 263)	(*n* = 198)
Sex, No. (%)			
Male	104(67.10)	171(65.02)	112(56.57)
Female	51(32.90)	92(34.98)	86(43.43)
Diagnosed age, years			
Median (IQR)	65.00 (58.00, 70.00)	63.00 (57.00, 69.00)	62.00 (56.00, 66.00)
BMI, kg/m^2^			
Median (IQR)	22.48 (20.57, 24.17)	21.69 (19.58, 23.45)	21.51 (19.96, 24.06)
Tumor location in pancreas, No. (%)			
Head/uncinate	53 (34.19)	81 (30.80)	70 (35.35)
Neck/proximal body	41 (26.45)	78 (29.66)	50 (25.25)
Distal body/tail	61 (39.35)	104 (39.54)	72 (36.36)
Loss data	0 (0)	0 (0)	6 (3.03)
Anatomical classification, No. (%)			
Resectable	5 (3.22)	21 (7.98)	NA
Borderline resectable	10 (6.45)	21 (7.98)	
Locally advanced	48 (30.97)	54 (20.53)	
Combined distant metastases	92(59.35)	167(63.50)	
Eighth edition AJCC stage, No. (%)			
I	4 (2.58)	11 (4.18)	9 (4.54)
II	19 (12.26)	40 (15.21)	36 (18.18)
III	40 (25.81)	45 (17.11)	46 (23.23)
IV	92 (59.35)	167 (63.50)	107 (54.04)
RECIST evaluation, No. (%)			
SD	88 (56.77)	192 (73.00)	106 (53.54)
PD	67 (43.23)	71 (27.00)	92 (46.46)

Abbreviation: BMI, body mass index.

Overall, the three cohorts, drawn from independent institutions and data sources, provided a suitable basis for model development and external validation.

### 2.2 Four Independent Prognostic Predictors Were Identified by Multivariable Cox Regression

Identification of clinically accessible predictors of OS is the foundation of the cPD score. We therefore performed sequential univariate and multivariable Cox regression analyses in the Training Cohort. All candidate demographic, clinical, laboratory, and imaging variables were first evaluated using univariate Cox proportional hazards regression. Variables reaching statistical significance (*p* < 0.05) were dichotomized using X‐tile software strictly within the Training Cohort to determine optimal cut‐off values, and were subsequently entered into the multivariable Cox model.

**FIGURE 2:**
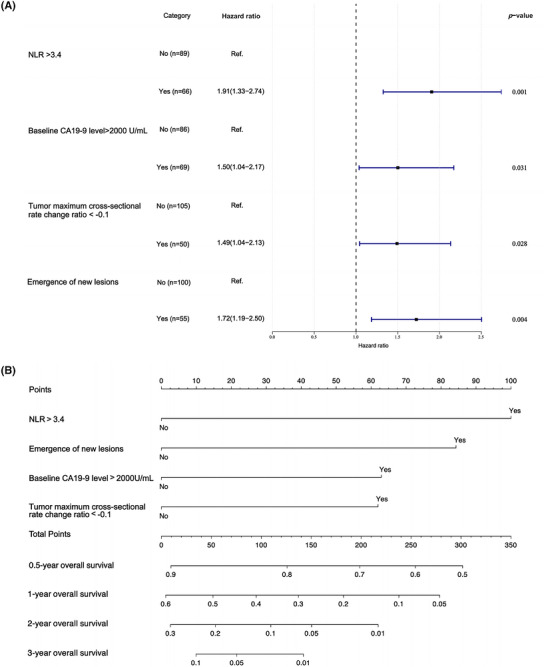
Construction of the cPD model based on multivariable Cox regression analysis in the Training Cohort. (A) Hazard ratio (HR) forest plot of the four independent prognostic predictors identified by multivariable Cox proportional hazards regression. (B) Constructed nomogram for individualized prognostic prediction at 6 months, 1 year, 2 years, and 3 years based on the four predictors.

Univariate analysis identified four significant prognostic factors for OS: NLR (hazard ratio [HR] = 1.05, 95% confidence interval [CI]: 1.01–1.10, *p* = 0.008), baseline CA19‐9 level (HR = 1.00, *p* = 0.001), tumor maximum cross‐sectional rate change ratio (HR = 0.78, 95% CI: 0.58–0.93, *p* = 0.010), and emergence of new lesions (HR = 1.96, 95% CI: 1.38–2.78, *p* < 0.001) (Table S1). X‐tile‐derived cut‐off values established within the Training Cohort were 3.4 for NLR, 2000 U/mL for CA19‐9, and −0.1 for tumor maximum cross‐sectional rate change ratio. Multivariable Cox regression confirmed all four as independent prognostic predictors with significant hazard ratios: NLR > 3.4 (HR = 1.91, 95% CI: 1.33–2.74, *p* = 0.001), baseline CA19‐9 > 2000 U/mL (HR = 1.50, 95% CI: 1.04–2.17, *p* = 0.031), tumor maximum cross‐sectional rate change ratio < −0.1 (HR = 1.49, 95% CI: 1.04–2.13, *p* = 0.028), and emergence of new lesions (HR = 1.72, 95% CI: 1.19–2.50, *p* = 0.004) (Figure 2A, Table S2). A nomogram integrating these four predictors was constructed for individualized OS prediction at 6 months, 1 year, 2 years, and 3 years (Figure 2B).

Taken together, four readily available clinical variables—NLR, baseline CA19‐9, tumor maximum cross‐sectional rate change ratio, and emergence of new lesions—were identified as independent prognostic predictors of OS, forming the foundation of the cPD scoring framework.

### 2.3 The cPD Score Was Developed With Three‐Group Classification Demonstrating Superior Discrimination

Translating the multivariable Cox model into a clinically accessible bedside tool requires an effective scoring system and an optimal risk stratification strategy. The cPD score was constructed by applying a linear transformation (HR × 2) to the four predictors identified by multivariable Cox regression, followed by a reverse scoring strategy: higher scores were assigned to the protective state (“No”) of each risk factor, such that patients with more favorable profiles accumulated higher total scores. The total cPD score therefore ranged from 8 (all four high‐risk features present) to 13 (no high‐risk features present). X‐tile software was then used—strictly within the Training Cohort—to determine optimal cut‐offs that classified patients into two‐ or three‐group prognostic categories. The discriminative performance of both classification strategies was evaluated using time‐dependent receiver operating characteristic (ROC) curves and dynamic area under the curve (AUC) analysis at 6 months, 1 year, 2 years, and 3 years, and model calibration was assessed using calibration plots based on four equally sized groups defined by predicted survival probability (Kaplan–Meier estimates within each group). The incremental predictive value of the integrated cPD score was further evaluated by comparing it with each of its four individual component variables.

**FIGURE 3:**
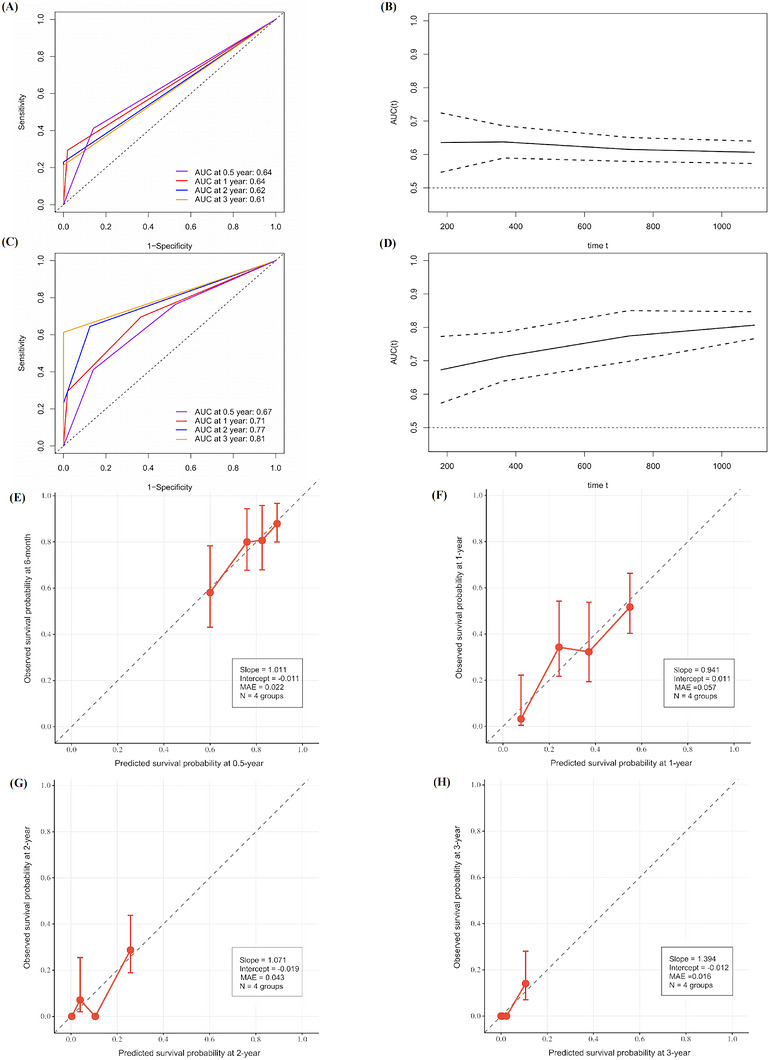
Risk stratification strategy and performance evaluation of the cPD model in the Training Cohort. (A) Time‐dependent ROC curves of the binary classification at four time points (0.5, 1, 2, and 3 years), with AUCs of 0.64, 0.64, 0.62, and 0.61, respectively. (B) AUC dynamics over time for the binary classification, with 95% confidence intervals (dashed lines). (C) Time‐dependent ROC curves of the three‐group classification at four time points, with AUCs of 0.67, 0.71, 0.77, and 0.81, respectively. (D) AUC dynamics over time for the three‐group classification, with 95% confidence intervals (dashed lines). (E–H) Calibration plots comparing model‐predicted and observed survival probabilities, with patients divided into four equally sized groups by predicted survival probability (*N* = 4 groups); calibration slope, intercept, and mean absolute error (MAE) are annotated at (E) 6 months, (F) 1 year, (G) 2 years, and (H) 3 years.

The detailed scoring assignments for each variable are presented in Table 2, and the frequency distribution of patients in the Training Cohort under both stratification schemes is summarized in Table S3. Comparison of model discrimination revealed that the three‐group classification (CP, score 8–9; PP, score 10–11; GP, score 12–13) consistently outperformed the binary classification across all time points: AUCs at 0.5, 1, 2, and 3 years were 0.67, 0.71, 0.77, and 0.81 for the three‐group classification versus 0.64, 0.64, 0.62, and 0.61 for the binary classification (Figure 3A–D). The three‐group classification also demonstrated excellent calibration at 6 months, 1 year, and 2 years, with calibration slopes near 1.0 (range: 0.94–1.07) and small mean absolute errors (MAE range: 0.022–0.057). At 3 years, the calibration slope was 1.394 (MAE 0.016), reflecting the limited number of patients remaining at risk at this time point in a disease with a median survival of less than 12 months (Figure 3E–H). In addition, the cPD composite score consistently outperformed each of its individual component variables (NLR, baseline CA19‐9, tumor maximum cross‐sectional rate change ratio, emergence of new lesions) across all evaluated time points, confirming the added discriminative value of the multivariable integration approach (Figure S1). The empirical validity of the LMS (long diameter × short diameter) bidimensional measurement underlying the tumor maximum cross‐sectional rate change ratio was further confirmed by its strong correlation with the standard RECIST unidimensional percentage change in both the Training Cohort (Pearson *r* = 0.849, *p* < 0.001) and Validation Cohort 1 (Pearson *r* = 0.846, *p* < 0.001) (Figure S2).

**TABLE 2:** Score for the Prognosis of PDAC After Chemotherapy.

Factors	Yes	No
Neutrophils/lymphocytes > 3.40	2	4
Baseline CA19‐9 level > 2000 U/mL	2	3
Tumor maximum cross‐sectional rate change ratio < −0.1	2	3
Emergence of new lesions	2	3

Collectively, the three‐group cPD classification (CP/PP/GP) provided superior discrimination and well‐calibrated survival prediction, outperforming both binary stratification and individual component variables, and was therefore adopted as the final cPD framework.

### 2.4 The cPD Groups Significantly Stratified OS in all Three Cohorts

Validation of the prognostic utility of the cPD three‐group classification is essential before clinical translation. We therefore examined OS differences across the CP, PP, and GP groups in the Training Cohort and two independent external validation cohorts using Kaplan–Meier survival curves and log‐rank tests. The distribution of RECIST‐defined SD and PD within each cPD group was additionally examined to evaluate the concordance between cPD stratification and conventional RECIST classification.

**FIGURE 4:**
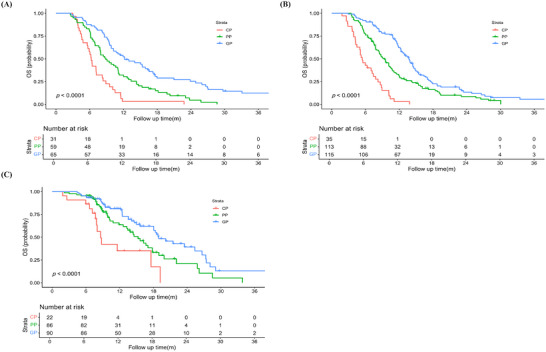
Overall survival stratified by the cPD groups in three cohorts. (A)The overall survival of Training Cohort. (B) The overall survival of Validation Cohort 1. (C) The overall survival of Validation Cohort 2.

In the Training Cohort, the three cPD groups demonstrated significantly different survival outcomes (log‐rank *p* < 0.0001), with median OS of 12.20 months for GP, 8.87 months for PP, and 6.27 months for CP (Figure 4A, Table S4). This stratification pattern was consistently reproduced in both validation cohorts: Validation Cohort 1 (log‐rank *p* < 0.0001; Figure 4B) and Validation Cohort 2 (log‐rank *p* < 0.0001; Figure 4C). Examination of the RECIST classification distribution across cPD groups revealed substantial discordance (Figure S3): in the Training Cohort, the GP group comprised 53 SD and 12 PD patients; the PP group comprised 31 SD and 28 PD; and the CP group comprised only 4 SD but 27 PD. Similar distribution patterns were observed in Validation Cohorts 1 and 2. Notably, RECIST‐defined PD patients were distributed across all three cPD groups, indicating that a single RECIST PD designation captured prognostically heterogeneous subsets that differed markedly in their underlying biological aggressiveness. These discordant cases—patients with favorable underlying prognostic profiles despite radiographic findings of progression—likely represent radiographic pseudoprogression and warrant particular attention in clinical decision‐making.

Overall, the cPD three‐group classification provided robust and reproducible OS stratification across all three cohorts and identified prognostically distinct subsets within RECIST‐defined SD and PD populations, supporting both its generalizability and its added clinical value beyond RECIST.

### 2.5 Continuing the Original Chemotherapy Regimen Benefited GP and PP Patients Despite RECIST‐Defined Progression

A key clinical question is whether cPD‐guided treatment decisions (continuation vs. alteration of the original chemotherapy regimen) translate into differential survival outcomes, particularly for patients classified as PD by RECIST. To address this, baseline comparability between Keep and Alter groups within each cPD subgroup was first assessed, including Eastern Cooperative Oncology Group (ECOG) performance status at both first chemotherapy initiation and first RECIST assessment, the age‐adjusted Charlson Comorbidity Index (CCI), and the proportion of patients experiencing ECOG deterioration during initial chemotherapy. Patients were then reclassified by treatment decision, and Kaplan–Meier survival analyses with HRs and 95% CI were performed for each cPD group in the Training Cohort and Validation Cohort 1.

**FIGURE 5:**
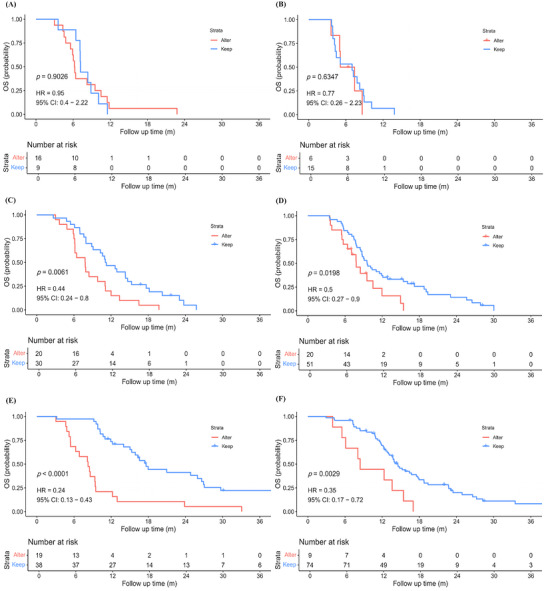
Overall survival in relation to the keep or alteration of therapeutic strategies within each cPD group. (A) CP group in Training Cohort. (B) CP group in Validation Cohort 1. (C) PP group in Training Cohort. (D) PP group in Validation Cohort 1. (E) GP group in Training Cohort. (F) GP group in Validation Cohort 1.

As detailed in Table S5, ECOG performance status (at both first chemotherapy and first assessment) and CCI did not differ significantly between Keep and Alter groups in either cohort (all *p* > 0.05). Notably, the proportion of patients experiencing ECOG deterioration during initial chemotherapy was virtually identical between the two groups (Training Cohort: 31.2% vs. 30.9%, *p* = 0.975; Validation Cohort 1: 17.1% vs 17.1%, *p* = 1.000), directly indicating that the treatment decision was not driven by ECOG dynamic changes and supporting the validity of the subsequent Keep versus Alter survival comparison. In both cohorts, the Keep strategy was associated with significantly better OS in the GP group (Training Cohort: HR = 0.24, 95% CI: 0.13–0.43, *p* < 0.0001; Validation Cohort 1: HR = 0.35, 95% CI: 0.17–0.72, *p* = 0.0029) (Figure 5E,F) and the PP group (Training Cohort: HR = 0.44, 95% CI: 0.24–0.80, *p* = 0.0061; Validation Cohort 1: HR = 0.50, 95% CI: 0.27–0.90, *p* = 0.0198) (Figure 5C,D). In contrast, no significant survival difference between Keep and Alter strategies was observed in the CP group (Training Cohort: HR = 0.95, 95% CI: 0.40–2.22, *p* = 0.9026; Validation Cohort 1: HR = 0.77, 95% CI: 0.26–2.23, *p* = 0.6347) (Figure 5A,B). The survival curves of the Keep and Alter arms intersected in the CP subgroup, suggesting that for patients with the most unfavorable cPD profile, OS outcomes were predominantly determined by the severity of the underlying disease rather than by subsequent treatment decisions—highlighting the urgent unmet need for novel therapeutic strategies in this high‐risk subgroup. The detailed median OS values for each subgroup are summarized in Table S6.

In summary, for GP and PP patients classified as PD by RECIST, continuing the original chemotherapy regimen provided significantly better survival than switching regimens, while CP patients did not benefit from either strategy. These findings demonstrate that the cPD score identifies clinically actionable subsets in whom RECIST‐based regimen alteration may be premature for some patients and insufficient for others.

### 2.6 Representative Imaging Cases Illustrate the Clinical Utility of cPD‐Guided Decision‐Making

The clinical utility of any prognostic model is best illustrated by representative real‐world cases. We therefore examined four patients classified as GP by the cPD model (cPD score = 12 points) but meeting PD criteria by RECIST guidelines at the first chemotherapy assessment. Two patients continued the original chemotherapy regimen (Keep strategy), and two underwent regimen alteration (Alter strategy). Their imaging features at baseline and at first RECIST assessment, cPD scores, treatment decisions, and survival outcomes were compared.

**FIGURE 6:**
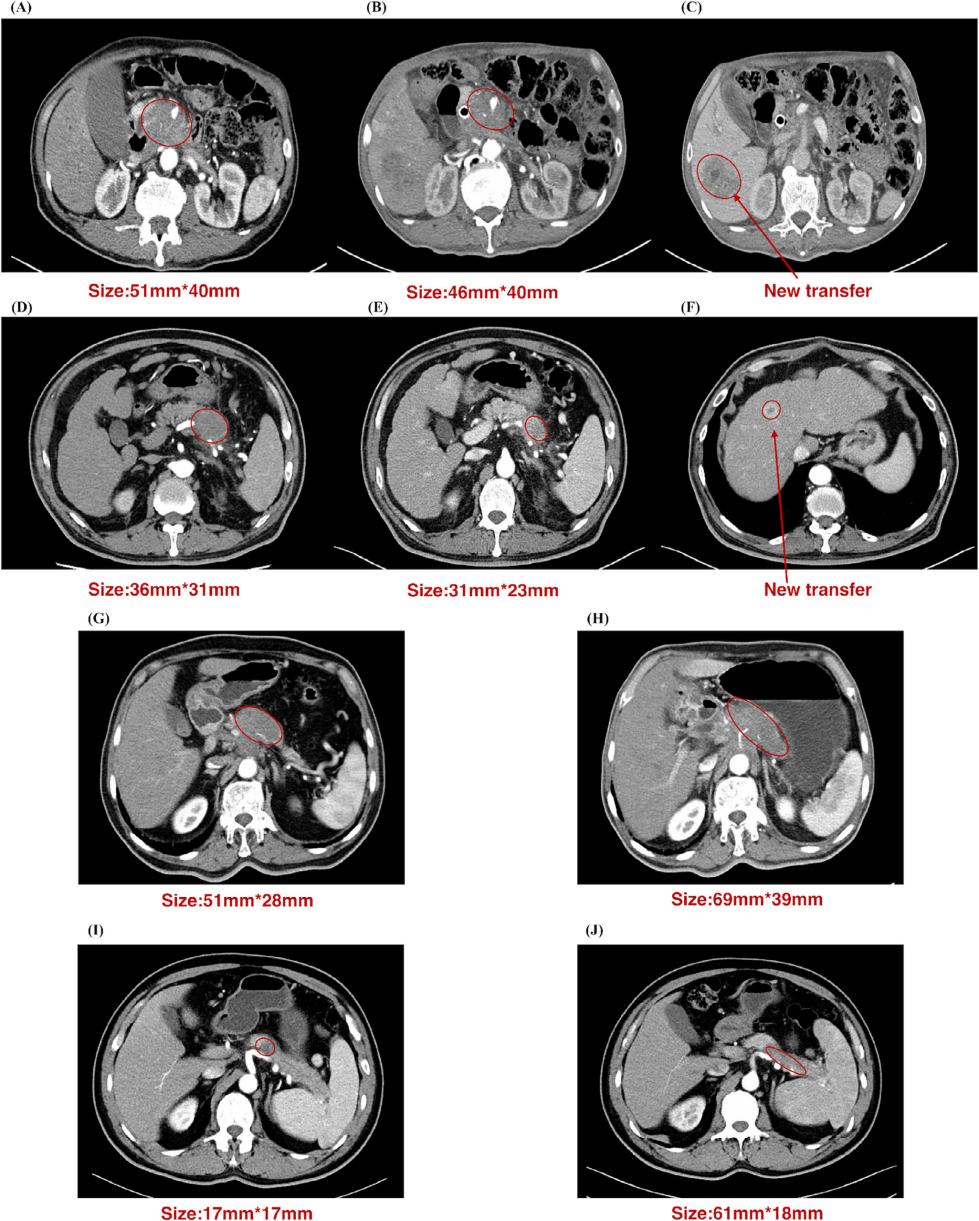
Representative imaging cases illustrating the application of the cPD model. (A–F) Type 1 cases—patients with emergence of new metastatic lesions during chemotherapy. Patient A: (A) Baseline CT image. (B) Follow‐up CT image showing the primary tumor. (C) Follow‐up CT image showing a newly emerged hepatic metastatic lesion. Patient B: (D) Baseline CT image. (E) Follow‐up CT image showing the primary tumor. (F) Follow‐up CT image showing a newly emerged hepatic metastatic lesion. (G–J) Type 2 cases—patients with substantial primary tumor enlargement during chemotherapy. Patient C: (G) Baseline CT image. (H) Follow‐up CT image showing marked tumor enlargement. Patient D: (I) Baseline CT image. (J) Follow‐up CT image showing marked tumor enlargement.

Two distinct imaging patterns of RECIST‐defined PD were identified within the GP group. Type 1—emergence of new metastatic lesions during chemotherapy—is illustrated by Patient A (Figure 6A–C) and Patient B (Figure 6D–F). Patient A developed new hepatic metastases during initial assessment but continued the original treatment based on cPD guidance and achieved a survival of 295 days. In contrast, Patient B presented with similar hepatic metastases, opted for treatment modification, and survived only 162 days. Type 2—substantial primary tumor enlargement during chemotherapy—is illustrated by Patient C (Figure 6G–H) and Patient D (Figure 6I–J). Patient C demonstrated > 20% tumor diameter increase at initial assessment but maintained the original regimen, achieving a survival of 420 days. Conversely, Patient D, with comparable tumor enlargement, elected to alter treatment and survived only 258 days. All four patients received a cPD score of 12 points (GP group), indicating that the cPD model would have recommended continuation of the original regimen for all four. The two patients who followed the cPD‐guided Keep strategy demonstrated markedly longer OS than the two patients who underwent RECIST‐driven regimen alteration, despite both subgroups meeting the same imaging definition of PD.

These case examples highlight that cPD‐guided treatment decisions can identify clinically meaningful subsets of patients in whom RECIST‐defined “progression” does not reflect true biological treatment failure, supporting the cPD score's added value beyond conventional radiographic assessment.

## 3 Discussion

In this study, we developed and externally validated the cPD score, a clinically accessible prognostic prediction model that integrates four readily available variables—NLR, baseline CA19‐9, tumor maximum cross‐sectional rate change ratio, and emergence of new lesions—to guide therapeutic decision‐making after the first chemotherapy assessment in PDAC. Chemotherapy response evaluation remains a controversial issue in current PDAC management, as the unique biological characteristics of PDAC—including peripheral tumor infiltration and post‐chemotherapy boundary obscuration—substantially limit the applicability of conventional RECIST for optimal response assessment [8, 22]. Post‐chemotherapy assessment should not rely exclusively on imaging results but should integrate tumor markers, the general condition of the patient, and other relevant factors. Our findings demonstrate that biological status (reflected by baseline CA19‐9), patient immune condition (reflected by NLR), tumor dynamic changes during chemotherapy, and the emergence of new lesions together provide a more comprehensive and accurate prognostic framework than any single indicator alone.

To address the limitations of RECIST unidimensional measurement in PDAC, our study introduces methodological enhancements by incorporating bidimensional tumor measurements (long diameter × short diameter, LMS). The empirical relationship between the LMS bidimensional change ratio and the standard RECIST unidimensional percentage change was validated by correlation analysis in both the Training Cohort and Validation Cohort 1 (Figure S2). The two metrics demonstrated strong positive correlation across both cohorts (Pearson *r* = 0.849 and 0.846, respectively; both *p* < 0.001), confirming methodological consistency with established RECIST principles. Importantly, the steeper‐than‐unity regression slope—together with the consistent observation that, among patients with LMS‐defined enlargement (>+10%), 52.2% (Training Cohort) and 62.9% (Validation Cohort 1) had RECIST unidimensional changes below the +20% PD threshold—demonstrates that LMS systematically captures a wider dynamic range of tumor changes than unidimensional measurement alone. This enhanced sensitivity is particularly relevant to PDAC, in which asymmetric tumor growth driven by dense stromal remodeling may not be fully reflected along the longest diameter alone, particularly when tumor enlargement occurs predominantly in the short‐axis dimension within the same axial cross‐sectional plane. The LMS bidimensional metric addresses this limitation by simultaneously incorporating both in‐plane diameters, providing a more complete surrogate of cross‐sectional tumor area.

Beyond imaging refinement, the cPD score's predictive power derives from its multidimensional integration of biological and radiographic parameters. A large meta‐analysis demonstrated that high NLR is associated with poor OS across a variety of solid tumors, and as an easily accessible and inexpensive inflammatory biomarker, NLR holds substantial value for clinical decision‐making [23]. The traditional serum marker CA19‐9 remains an important indicator for diagnosis, staging, prognosis prediction, and treatment response assessment in PDAC [24]. However, single biomarker‐based approaches, whether NLR or CA19‐9 alone, consistently yield inferior discriminative performance compared with the integrated cPD composite score across all evaluated time points (Figure S1). This finding empirically supports the conceptual rationale that no single biomarker or imaging parameter can fully capture the multidimensional complexity of PDAC treatment response, and that integration of complementary prognostic dimensions—immune (NLR), tumor biology (CA19‐9), dynamic morphology (LMS), and metastatic emergence—provides the prognostic precision required for clinical decision‐making.

The cPD model utilizes clinically readily available metrics, is convenient and feasible to apply at the bedside, and accurately predicts patient survival outcomes. By categorizing patients into three distinct prognostic groups (CP, PP, GP), the model achieves precise risk stratification across all three cohorts: the median survival in the GP group was 12.20, 13.70, and 18.93 months in the Training Cohort, Validation Cohort 1, and Validation Cohort 2, respectively, while the median survival in the CP group was significantly shorter—6.27, 5.43, and 8.60 months, respectively. The robust differentiation of survival outcomes across all three independent cohorts, with consistent ordering of GP > PP > CP and significant log‐rank tests (all *p* < 0.0001), confirms the generalizability and prognostic validity of the cPD model.

Importantly, the cPD score not only stratifies prognosis but also informs actionable therapeutic decisions. Treatment decision analysis based on the cPD score revealed clinically meaningful implications: in the GP and PP groups, patients who continued the original chemotherapy regimen demonstrated significantly superior OS compared with those who altered their treatment regimen, suggesting that these patients were responding well to the current treatment and benefit from continuation. Notably, this survival benefit persisted even among patients classified as PD by RECIST, indicating that RECIST‐defined “progression” does not uniformly reflect true biological treatment failure in PDAC. In contrast, the CP subgroup (Figure 5A,B) showed no significant survival difference between Keep and Alter strategies (Training Cohort: *p* = 0.9026, HR = 0.95; Validation Cohort 1: *p* = 0.6347, HR = 0.77), suggesting that for patients with the most unfavorable cPD profile, OS outcomes are predominantly determined by the severity of the underlying disease rather than by subsequent treatment decisions, highlighting the urgent unmet clinical need for novel therapeutic strategies in this high‐risk subgroup.

The observation that RECIST‐defined “progression” does not uniformly reflect biological treatment failure can be mechanistically explained by the phenomenon of radiographic pseudoprogression—defined as apparent radiographic tumor enlargement that does not reflect true disease progression—which represents a significant yet underrecognized challenge in PDAC chemotherapy management [22]. This phenomenon is mechanistically rooted in the unique biology of PDAC, characterized by an exceptionally dense desmoplastic stroma that may comprise up to 80% of the tumor mass. Dense fibrotic stroma frequently persists or even progresses after cytotoxic chemotherapy, producing apparent tumor enlargement on cross‐sectional imaging without corresponding viable cancer cell proliferation. It is important to distinguish this chemotherapy‐related stromal pseudoprogression from immune‐related pseudoprogression observed during immune checkpoint inhibitor therapy, which arises from intratumoral lymphocytic infiltration and inflammation‐associated edema, and is monitored using the dedicated iRECIST framework [25]; in contrast, radiographic pseudoprogression in PDAC chemotherapy reflects stromal and fibrotic responses rather than active immune‐mediated processes, and currently lacks a dedicated assessment framework. While the impact of PDAC stromal fibrosis on radiographic tumor size measurement has been recognized in the resectable/neoadjuvant setting [22], its quantitative prevalence in advanced PDAC undergoing palliative chemotherapy—and its impact on therapeutic decision‐making—has not been previously characterized in a systematic, multicohort manner. A recent first case report of biochemical pseudoprogression in chemotherapy‐treated PDAC (reporting CA19‐9 elevation followed by decline) addressed only a serological manifestation, whereas radiographic pseudoprogression in PDAC chemotherapy remains inadequately characterized [26].

The clinical relevance of recognizing radiographic pseudoprogression in PDAC chemotherapy is substantial: misinterpretation as true progression may lead to premature treatment alteration or discontinuation, depriving patients of an effective ongoing regimen. Analysis of SD/PD distribution across all cohorts revealed clinically meaningful proportions of GP‐classified patients meeting PD criteria by RECIST: 18.46% (12/65) in the Training Cohort, 12.17% (14/115) in Validation Cohort 1, and 36.67% (33/90) in Validation Cohort 2. This suggests that a clinically meaningful proportion of patients may have experienced radiographic pseudoprogression during treatment, potentially leading to premature therapy alteration or treatment discontinuation if managed solely on the basis of RECIST classification. Rather than attempting to directly distinguish radiographic pseudoprogression from true progression on imaging—which remains technically challenging—the cPD score circumvents this difficulty by integrating multiple independent prognostic factors (NLR, CA19‐9, radiological dynamics, and new lesion emergence) into a composite framework that identifies patients with favorable underlying prognosis despite RECIST‐defined progression, thereby informing more nuanced therapeutic decision‐making in this clinically challenging context.

It is important to contextualize the cPD score within the landscape of existing prognostic scoring systems for PDAC. The mGPS, based on C‐reactive protein and albumin levels, is among the most extensively validated inflammation‐based prognostic markers in PDAC, with meta‐analytic evidence confirming its association with OS [27]. However, mGPS primarily reflects host systemic inflammation and nutritional status rather than tumor‐specific treatment response, limiting its utility for guiding post‐chemotherapy therapeutic decisions. More directly comparable to the cPD model, the scoring system proposed by Tanaka et al. integrates tumor shrinkage, tumor density, and post‐chemotherapy CA19‐9 to predict outcomes after FOLFIRINOX induction in locally advanced pancreatic cancer [21]; however, this model was limited to resected LAPC patients (*n* = 62) without external validation and did not incorporate inflammatory markers or provide direct therapeutic recommendations. Research on ETS as a prognostic predictor, including the exploratory analysis of JCOG1407, has demonstrated the prognostic value of radiological response in PDAC [20], but ETS remains a single imaging metric without integration of biological parameters. In contrast, the cPD score uniquely combines biological and imaging‐based variables at the first post‐chemotherapy evaluation, and—critically—translates prognostic stratification into actionable therapeutic guidance (keep vs. alter therapy), a feature absent from all aforementioned models. The cPD score thus acts as a dynamic, multidimensional, yet highly accessible bedside tool that bridges the gap by combining post‐treatment morphological responses with baseline systemic biological profiles, requiring no advanced software for daily clinical use. The multi‐institutional validation across 616 patients further supports its generalizability. Nevertheless, future model refinement may benefit from incorporating additional parameters such as ECOG performance status, tumor density changes, or emerging molecular biomarkers to further enhance predictive accuracy.

Returning to the unmet need of the CP subgroup identified earlier, recent advances in molecularly targeted therapies and immunotherapy offer promising new directions for this high‐risk population. Daraxonrasib (RMC‐6236), an oral RAS(ON) multi‐selective inhibitor, recently demonstrated substantial antitumor activity in patients with previously treated metastatic PDAC harboring RAS mutations, leading to U.S. Food and Drug Administration Breakthrough Therapy Designation in 2025 [28]. Considering that more than 90% of PDAC tumors harbor activating RAS mutations, RAS(ON) inhibition may provide a meaningful therapeutic option for CP‐classified patients who do not benefit from conventional chemotherapy modification. In addition, personalized mRNA neoantigen vaccines such as autogene cevumeran have shown promising results in adjuvant PDAC trials, generating durable polyfunctional CD8+ T‐cell responses and correlating with delayed disease recurrence [29, 30]. Integration of the cPD score with such emerging therapies—either by prioritizing CP‐classified patients for clinical trials of novel agents or by combining cPD‐guided decisions with molecularly targeted approaches—may further refine personalized therapy in advanced PDAC and improve outcomes in this most vulnerable subgroup.

Several limitations of this study should be acknowledged. First, retrospective analyses are inevitably subject to selection bias, which we minimized through standardized inclusion criteria and multicenter validation; nonetheless, prospective cohorts are needed for definitive validation, and a prospective trial directly comparing cPD‐guided versus RECIST‐guided treatment decisions represents an important and necessary direction for future research. Second, there was heterogeneity in the follow‐up period between the CPDC database and the five cooperative medical institutions in Zhejiang Province, but loss‐of‐visit bias was effectively controlled by requiring at least 6 months of follow‐up after diagnosis as part of the inclusion criteria. Third, fluorodeoxyglucose positron emission tomography/computed tomography (FDG‐PET/CT) was not routinely available for all patients, limiting our ability to incorporate metabolic response markers into the cPD framework. Fourth, CA19‐9 secretion is dependent on Lewis antigen expression; patients who are Lewis antigen–negative are constitutively unable to produce CA19‐9 (CA19‐9 non‐producers, defined as baseline CA19‐9< 2 U/mL). In the present study, CA19‐9 non‐producers comprised 11 of 155 patients (7.1%) in the Training Cohort, 16 of 263 patients (6.1%) in Validation Cohort 1, and 14 of 198 patients (7.1%) in Validation Cohort 2, consistent with reported prevalence estimates in Asian populations [15, 31]. These patients were retained in the analysis without exclusion or imputation to preserve the real‐world representation of our cohorts. Given that the cPD model applies a high binary risk threshold of 2000 U/mL for CA19‐9, CA19‐9 non‐producers were naturally categorized into the low‐risk category for this variable and assigned a score of 3 points, which is clinically appropriate because non‐producer status per se does not confer adverse prognosis. While this biological non‐secretion might theoretically underestimate the risk profile of this small subset of patients regarding the CA19‐9 variable alone, the multidimensional composite nature of the cPD score mitigates this limitation: the inclusion of independent parameters such as NLR, tumor morphological changes, and the emergence of new lesions ensures that the overall prognostic evaluation remains robust even when CA19‐9 is biologically uninformative. Fifth, the cPD scoring system was designed as a single time‐point assessment tool applied at the first RECIST evaluation, and longitudinal re‐measurement of cPD scores following treatment modification was not incorporated into the study design. Consequently, dynamic changes in cPD scores across sequential treatment cycles could not be examined. Future prospective studies should incorporate serial cPD score measurements to evaluate the model's responsiveness to therapeutic modification and its utility in guiding ongoing treatment decisions beyond the initial assessment.

## 4 Conclusion

In conclusion, we developed and externally validated the cPD score, a clinically accessible prognostic prediction model integrating four readily available variables—NLR, baseline CA19‐9, tumor maximum cross‐sectional rate change ratio, and emergence of new lesions—to guide therapeutic decision‐making after the first chemotherapy assessment in advanced PDAC. The cPD score stratifies patients into three prognostic groups (CP, PP, GP) with robust and reproducible survival differentiation across one training and two independent external validation cohorts comprising 616 patients in total. Critically, the cPD score provides actionable therapeutic guidance: patients in the GP and PP groups derive significant survival benefit from continuing the original chemotherapy regimen even when classified as PD by RECIST, whereas patients in the CP group represent an urgent unmet clinical need for novel therapeutic approaches. By integrating biological and imaging dimensions into a single, multidimensional, bedside‐applicable scoring framework, the cPD score offers oncologists a practical tool to refine post‐chemotherapy decision‐making in PDAC and to better stratify management of this lethal malignancy. Further prospective validation and integration with emerging molecularly targeted therapies will be essential to fully establish the clinical utility of this scoring system.

## 5 Materials and Methods

### 5.1 Study Design and Patient Selection

This was a multicenter retrospective cohort study involving five major PDAC treatment centers in Zhejiang Province—the Second Affiliated Hospital of Zhejiang University School of Medicine, the First Affiliated Hospital of Wenzhou Medical University, Zhejiang Cancer Hospital, Huzhou Central Hospital, and the Fourth Affiliated Hospital of Zhejiang University School of Medicine—and the CPDC database. The study was conducted in accordance with the Declaration of Helsinki and was approved by the Institutional Review Board of each participating center. Written informed consent was waived owing to the retrospective nature of the study.

Patients were assigned to three cohorts based on the source institution: the Training Cohort (SAHZU, *n* = 155), used for cPD score development; Validation Cohort 1 (four Zhejiang Province institutions, *n* = 263); and Validation Cohort 2 (CPDC database, *n* = 198). The same inclusion criteria were applied to all three cohorts: (1) histopathologically confirmed PDAC; (2) treated with chemotherapy as initial systemic therapy; (3) classified as SD or PD at the first radiological assessment using RECIST 1.1 criteria; (4) availability of complete baseline and first‐assessment imaging data, laboratory biomarkers, and clinical records; and (5) minimum follow‐up duration of 6 months after diagnosis. Patients with PR or CR at first assessment were excluded.

### 5.2 Clinical, Radiological, and Biological Features

Clinical characteristics were systematically extracted from patients' medical records. Tumor staging followed the 8th edition of the AJCC staging system. ECOG performance status was recorded at chemotherapy initiation and first RECIST assessment. The age‐adjusted CCI was calculated for each patient using the standard weighted comorbidity scoring algorithm with an additional point added per decade of age above 40 years [32]. Imaging was independently evaluated by two experienced radiologists according to RECIST 1.1. To better capture morphological changes in PDAC, we additionally introduced a bidimensional measurement metric (LMS = long diameter × short diameter) and defined the tumor maximum cross‐sectional rate change ratio as $1-\frac{\text{LMSe}}{\text{LMSb}}$. Emergence of new lesions was defined as any new neoplastic lesion at first RECIST assessment, absent at baseline. The NLR was calculated as: $NLR=\frac{\text{Neutrophils}}{\text{Lymphocytes}}$. Baseline CA19‐9 was measured within 7 days before chemotherapy initiation; CA19‐9 non‐producers (< 2 U/mL) were retained without exclusion or imputation.

### 5.3 Definitions of "Keep" and "Alter" Treatment Strategies

To enable analysis of treatment decision‐making, each patient's subsequent treatment strategy after the first RECIST assessment was categorized into one of two operationally defined groups:

“Keep” strategy: Continuation of the original chemotherapy regimen (including the same drugs at the same or modified doses) without switching to a different regimen. Dose adjustments due to toxicity or supportive modifications did not qualify as “Alter.”

“Alter” strategy: Switching to a different chemotherapy regimen, defined as the substitution of at least one core cytotoxic agent with a different cytotoxic agent from a distinct drug class (e.g., switching from gemcitabine‐based to FOLFIRINOX or vice versa, or adding/removing platinum‐based agents that fundamentally change the regimen backbone).

Patients who discontinued chemotherapy entirely (e.g., due to clinical deterioration or patient choice) without initiating a new regimen were excluded from the Keep‐versus‐Alter analysis.

### 5.4 Outcome and Follow‐Up

The primary outcome was OS, defined as the time interval between the date of the baseline CT imaging and death from any cause. Patients were followed until death or the study end date (November 22, 2024). Data were censored at the date of last contact for patients who were lost to follow‐up or remained alive at the study end date.

### 5.5 Statistical Analysis

Univariate and multivariable Cox proportional hazards regression were used to identify independent prognostic predictors in the Training Cohort. Continuous variables were dichotomized using X‐tile software (version 3.6.1) applied exclusively to the Training Cohort, and the derived cutoffs were applied without modification to both validation cohorts. The cPD score was constructed by integer transformation of the multivariable Cox hazard ratios followed by a reverse scoring strategy, with patients stratified into CP, PP, and GP groups. Following the TRIPOD reporting guideline, model performance was evaluated using discrimination (time‐dependent ROC analysis at 6 months, 1 year, 2 years, and 3 years) and calibration (calibration plots and mean absolute error). Survival analysis used Kaplan–Meier estimation with log‐rank tests, and treatment decision impact (Keep versus Alter) was assessed within each cPD subgroup. Two‐tailed *p* < 0.05 was considered statistically significant. Detailed statistical methods can be found in the Supporting Information.

### 5.6 Software

Baseline characteristics analysis was performed using SPSS Statistics (version 27.0). Subsequent prognostic modeling, survival analysis, and visualization were performed using R (version 4.3.3) with the survival, survminer, rms, timeROC, forestplot, ggplot2, and ggpubr packages. X‐tile software (version 3.6.1) was used to determine optimal cut‐off values for continuous variables.

Detailed materials and methods can be found in the Supporting Information.

## Author Contributions

Conception and design: Yuan Ding, Weilin Wang, Zhongquan Sun, and Yixin Zhang. Collection and assembly of data: Yixin Zhang, Zhongquan Sun, Hanqi Yu, Xiaochang Wu, Changlin Zou, Linping Dong, Qi Xu, Jiali Ji, Haoliang He, Yufeng Zhong, and Xin Han. Data analysis: Zhongquan Sun, Yixin Zhang, Hongfan Ding, Hanqi Yu, and Jiali Ji. Manuscript writing: Yixin Zhang, Zhongquan Sun, Hongfan Ding, Hanqi Yu, Changlin Zou, and Jiali Ji. Final approval of manuscript: All authors. All authors have read and approved the final manuscript.

## Funding

This research was supported by Key Research and Development Program of Zhejiang Province (No. 2024C03143).

## Ethics Statement

The study was in accordance with the ethical guidelines of the 1975 Declaration of Helsinki. Ethical approval was obtained from the Ethics Committee of the Second Affiliated Hospital of Zhejiang University (2024‐1437), the Ethics Committee of the First Affiliated Hospital of Wenzhou Medical University (2025‐0409‐4), the Ethics Committee of Zhejiang Cancer Hospital (IRB‐2025‐92), the Ethics Committee of Huzhou Central Hospital (202503015‐01), and the Ethics Committee of the Fourth Affiliated Hospital of Zhejiang University (2025‐R098).

## Conflicts of Interest

The authors declare no conflicts of interest.

## Supporting information




**Supporting Information file 1**: mco270903‐sup‐0001‐SuppMat.docx

## Data Availability

All data generated during this study are included in this published article and its Supporting Information files. Anonymized data will be made available from the corresponding author upon reasonable request.
