# Supplementary material for: A Chemotherapy Progression Decision Score for Treatment Selection After Initial Assessment in Pancreatic Ductal Adenocarcinoma
Source: MedComm (2020). 2026 Aug 6;7(8):e70903. doi: 10.1002/mco2.70903 (PMC13448143; doi:10.1002/mco2.70903)
Supplement: Supplementary file 1 [file MCO2-7-e70903-s001.docx]

**Supplementary Information file**

**Title page**

**Title****:** A Chemotherapy Progression Decision Score for Treatment Selection after Initial Assessment in Pancreatic Ductal Adenocarcinoma

**Authors:** Yixin Zhang ^1,2,3,4,5,6#^, Zhongquan Sun^1,2,3,4,5,6^^#^, Hongfan Ding ^1,2,3,4,5,6^^#^, Changlin Zou^7#^, Linping Dong^8^,Qi Xu^9^, Jiali Ji ^1,2,3,4,5,6^, Xin Han ^1,2,3,4,5,6^, Hanqi Yu^1,2,3,4,5,6^, Daqian Xu^10^, Gaopeng Li^11^, Yuhao Wang^10^, Haoliang He^1,2,3,4,5,6^, Yufeng Zhong^1,2,3,4,5,6^, Xiaochang Wu^12*^, Jieer Ying^9*^, Weilin Wang^1,2,3,4,5,6*^, Yuan Ding^1,2,3,4,5,6^^*^

1 Department of Hepatobiliary and Pancreatic Surgery, The Second Affiliated Hospital, Zhejiang University School of Medicine, Hangzhou, Zhejiang, China

2 Key Laboratory of Precision Diagnosis and Treatment for Hepatobiliary and Pancreatic Tumor of Zhejiang Province, Hangzhou, Zhejiang, China

3 Research Center of Diagnosis and Treatment Technology for Hepatocellular Carcinoma of Zhejiang Province, Hangzhou, Zhejiang, China

4 Center for Medical Research and Innovation in Digestive System Tumors, Ministry of Education, China

5 Cancer Center, Zhejiang University, Hangzhou, Zhejiang, China

6 ZJU-Pujian Research & Development Center of Medical Artificial Intelligence for Hepatobiliary and Pancreatic Disease, Hangzhou, Zhejiang, China

7 Department of Radiotherapy and Chemotherapy, the First Affiliated Hospital of Wenzhou Medical University, Wenzhou, Zhejiang, China

8 Department of Hepatobiliary and Pancreatic Surgery, The Fourth Affiliated Hospital, Zhejiang University School of Medicine, Yiwu, Zhejiang, China

9 Department of Hepato-Pancreato-Biliary & Gastric Medical Oncology, Zhejiang Cancer Hospital, Hangzhou Institute of Medicine (HIM), Chinese Academy of Sciences, Hangzhou, Zhejiang, China

10Zhejiang Provincial Key Laboratory of Pancreatic Disease, The First Affiliated Hospital, Institute of Translational Medicine, Zhejiang University School of Medicine, Zhejiang University, Hangzhou, China.

11Department of Colorectal Surgery and Oncology of the Second Affiliated Hospital and Institute of Translational Medicine, Zhejiang University School of Medicine, Hangzhou, China

12 Department of Hepatobiliary Surgery, Huzhou Central Hospital, Zhejiang University Huzhou Hospital, Huzhou, Zhejiang, China

^#^These authors contributed equally to this work.

The authors declare no potential conflicts of interest.

**Corresponding to:** Yuan Ding, Department of Hepatobiliary and Pancreatic Surgery, The Second Affiliated Hospital, Zhejiang University School of Medicine, No. 88 Jiefang Road, Hangzhou, Zhejiang, China, 310009, E-mail: [dingyuan@zju.edu.cn](mailto:dingyuan@zju.edu.cn), ORCID: 0000-0002-3840-9886. Weilin Wang, Department of Hepatobiliary and Pancreatic Surgery, The Second Affiliated Hospital, Zhejiang University School of Medicine, No. 88 Jiefang Road, Hangzhou, Zhejiang, China, 310009, E-mail: [wam@zju.edu.cn](mailto:wam@zju.edu.cn), ORCID: 0000-0001-9432-2649. Jieer Ying, Department of Hepato-Pancreato-Biliary & Gastric Medical Oncology, Zhejiang Cancer Hospital, Hangzhou Institute of Medicine (HIM), Chinese Academy of Sciences, Hangzhou, Zhejiang, China, E-mail: [jieerying@aliyun.com](mailto:jieerying@aliyun.com).And to Xiaochang Wu, Department of Hepatobiliary Surgery, Huzhou Central Hospital, Zhejiang University Huzhou Hospital, Huzhou, Zhejiang, China, E-mail: [wuxiaochang88@sina.com](mailto:wuxiaochang88@sina.com).

**S1. Detailed clinical, radiological, and biological assessments**

Charlson Comorbidity Index (CCI) calculation. The age-adjusted CCI was calculated for each patient using the standard 19-item weighted scoring algorithm, including age adjustment (+1 for each decade of age over 40 years) and differential weighting for metastatic (+6) versus non-metastatic (+2) solid tumor based on baseline metastatic status. This comprehensive comorbidity measure was used to assess baseline comparability between treatment-decision groups.

Definition of CA19-9 non-producers. CA19-9 secretion is dependent on Lewis antigen expression; patients who are Lewis antigen–negative are constitutively unable to produce CA19-9 (CA19-9 non-producers, defined as baseline CA19-9 < 2 U/mL). CA19-9 non-producers were retained in the analysis without exclusion or imputation. Given that the cPD model applies a high binary risk threshold of 2000 U/mL for CA19-9, CA19-9 non-producers were naturally categorized into the low-risk category for this variable.

Imaging assessment details. Imaging evaluations were performed independently by two experienced radiologists, blinded to treatment decisions and outcomes. The measurements of target lesions, non-target lesions, and new lesions strictly followed RECIST 1.1 criteria. The bidimensional measurement metric was defined as:

LMS = long diameter × short diameter of the target lesion

Tumor maximum cross-sectional rate change ratio = 1-$\frac{LMSe}{\mathrm{LMS}b}$ ,

where LMSₑ denotes the LMS at the first RECIST evaluation, and LMS_b_ denotes the LMS at baseline.

Biological features. Baseline laboratory data—including absolute neutrophil count, lymphocyte count, alanine aminotransferase (ALT), aspartate aminotransferase (AST), albumin, prothrombin time (PT), activated partial thromboplastin time (APTT), international normalized ratio (INR), and CA19-9—were collected within seven days before the initiation of first-line chemotherapy.

**S2. Detailed Statistical Methods**

S2.1 Descriptive statistics

Baseline characteristics were summarized using descriptive statistics. Categorical variables were presented as frequency counts and percentages (n, %), and continuous variables as median values with interquartile ranges (IQR). Baseline characteristics were compared descriptively across the three cohorts.

S2.2 Variable selection and cut-off determination

All candidate clinical, laboratory, and imaging variables were first evaluated using univariate Cox proportional hazards regression in the Training Cohort. Variables reaching p < 0.05 in univariate analysis were considered for inclusion in the multivariable Cox model. Continuous variables retained for multivariable analysis were dichotomized using X-tile software (version 3.6.1; Yale University, New Haven, CT, USA)—a compact bioinformatics tool developed for optimizing biomarker cut-off values particularly suited for survival data analysis—applied exclusively to the Training Cohort to determine optimal cut-off values that maximized OS stratification. The X-tile-derived cut-offs were then applied without modification to both validation cohorts; no additional cut-off derivation was performed in the validation cohorts.

S2.3 Baseline comparability between Keep and Alter groups

To assess the comparability of baseline characteristics between the Keep and Alter treatment-decision groups within each cPD subgroup, the Mann–Whitney U test was used for ECOG performance status (at both first chemotherapy and first assessment), the independent t-test was used for CCI, and the χ² test was used for the proportion of patients experiencing ECOG worsening during initial chemotherapy.

S2.4 Multivariable Cox modeling and nomogram construction

Multivariable Cox proportional hazards regression was performed using the dichotomized variables to identify independent prognostic predictors. The proportional hazards assumption was assessed using Schoenfeld residuals. A nomogram for individualized OS prediction at 6 months, 1 year, 2 years, and 3 years was constructed based on the multivariable Cox model.

S2.5 cPD score construction

To translate the multivariable Cox model into a practical bedside tool, an integer-based scoring system was developed. We first applied a linear transformation by multiplying each hazard ratio (HR) by 2 and rounding to the nearest integer, producing weighted point values proportional to each variable's prognostic contribution. To align the final scoring system with the intuitive clinical convention that higher scores reflect better prognosis, a reverse scoring strategy was then applied: for each binary risk factor, the full integer weight was assigned to the protective state ("No"), while a baseline score of 2 points was assigned to the high-risk state ("Yes"). Specifically, the score assignments were as follows:

NLR > 3.4 (HR = 1.91): Yes = 2, No = 4

Baseline CA19-9 > 2000 U/mL (HR = 1.50): Yes = 2, No = 3

Tumor maximum cross-sectional rate change ratio <−0.1 (HR = 1.49): Yes = 2, No = 3

Emergence of new lesions (HR = 1.72): Yes = 2, No = 3

The total cPD score for each patient was calculated by summing the individual variable scores. A patient presenting none of the high-risk features therefore accumulates the maximum total score of 13 points (GP group), whereas a patient fulfilling all four risk criteria receives the minimum score of 8 points (CP group).

S2.6 Risk group stratification

For optimal risk stratification, X-tile software was again employed within the Training Cohort to determine the most appropriate cut-off values, classifying patients into two- or three-group prognostic categories. The classification system yielding the higher AUC in time-dependent ROC analysis was selected as the final cPD score model. Based on this selection criterion, the three-group classification was adopted as the final cPD framework: Critical Prognosis (CP, score 8–9), Poor Prognosis (PP, score 10–11), and Good Prognosis (GP, score 12–13).

S2.7 Model performance assessment

Following the TRIPOD reporting guideline, model performance was evaluated using two complementary domains: discrimination and calibration. The predictive performance of the finalized cPD score model was evaluated through time-dependent receiver operating characteristic (time-ROC) analysis at four clinically meaningful milestones—six months, one year, two years, and three years. These time points pertain exclusively to the model evaluation phase and are distinct from the model derivation phase in which the global HRs were estimated. The incremental discriminative value of the cPD composite score over each individual component variable was further evaluated by comparative time-dependent ROC analysis. Calibration was assessed by comparing predicted survival probabilities (derived from the multivariable Cox model) with observed Kaplan-Meier survival estimates across four equally sized groups of predicted survival; calibration plots, calibration slopes, intercepts, and mean absolute error (MAE) were reported at the four time points.

S2.8 Survival analysis and treatment decision evaluation

Kaplan-Meier survival curves were used to estimate OS in each cPD group across the three cohorts, with between-group differences compared using the log-rank test. To evaluate the impact of treatment strategy (Keep vs Alter) on survival, patients within each cPD group were reclassified according to their post-assessment treatment decision. Kaplan-Meier survival analysis with log-rank tests and Cox-derived HRs with 95% confidence intervals was performed within each cPD group.

Figure S1. Time-ROC curves comparing the predictive performance of individual component variables and the cPD score in the Training Cohort. (A)6-month time-ROC curves. (B)1-year time-ROC curves. (C)2-year time-ROC curves. (D)3-year time-ROC curves.

Figure S2. Correlation between the LMS bidimensional change ratio and the standard RECIST unidimensional percentage change. (A)Training Cohort. (B)Validation Cohort 1.

Figure S3. SD/PD distribution analysis: frequency distribution of RECIST-defined progressive disease (PD) and stable disease (SD) within the cPD groups (GP, PP, CP) across the three cohorts.

| **Table S1. Univariate Cox Proportional Hazards Regression Analysis of Factors Associated with Overall Survival** | | |
| --- | --- | --- |
| **Factors** | **Univariate** | |
|  | **HR (95% CI)** | ***p*** |
| Sex |  |  |
| Male  Female | Ref.  0.86(0.61-1.22) | 0.388 |
| Diagnosed age, years |  |  |
| < 60  ≥ 60 | Ref.  0.94(0.65-1.34) | 0.716 |
| BMI |  |  |
| Normal(18.5≤BMI<24)  Abnormal | Ref.  0.99(0.69-1.44) | 0.965 |
| Tumor location in pancreas |  |  |
| Head/uncinate  Neck/proximal body  Distal body/tail | Ref.  0.82(0.54-1.26)  1.19(0.82-1.73) | 0.367  0.370 |
| Anatomical classification |  |  |
| Resectable  Borderline resectable  Locally advanced  Combined distant metastases | Ref.  1.89(0.63-5.66)  0.93(0.37-2.37)  1.76(0.71-4.36) | 0.254  0.888  0.219 |
| 8th edition AJCC stage |  |  |
| Ⅰ  Ⅱ  Ⅲ  Ⅳ | Ref.  1.18(0.40-3.50)  1.10(0.39-3.09)  1.92(0.70-5.24) | 0.768  0.861  0.205 |
| NLR | 1.05(1.01-1.10) | ***0.008*** |
| ALT | 1.00(1.00-1.00) | 0.279 |
| AST | 1.00(1.00-1.00) | 0.416 |
| Albumin | 0.99(0.95-1.03) | 0.567 |
| PT | 1.12(0.91-1.38) | 0.283 |
| APTT | 1.00(0.95-1.05) | 0.995 |
| INR | 3.44(0.42-28.25) | 0.249 |
| Baseline CA19-9 level | 1.00(1.00-1.00) | ***0.001*** |
| Tumor maximum cross-sectional rate change ratio (long diameter*short diameter) | 0.78(0.58-0.93) | ***0.010*** |
| Emergence of new lesions |  |  |
| No  Yes | Ref.  1.96(1.38-2.78) | ***<0.001*** |

| **Table S2. Univariate and** **Multivariate Cox Proportional Hazards Regression Analysis of** **Factors (after x-tile calculation) Associated with Overall Survival** | | | | |
| --- | --- | --- | --- | --- |
| **Factors** | **Univariate** | | **Multivariate** | |
|  | **HR (95% CI)** | ***p*** | **HR (95% CI)** | ***p*** |
| NLR >3.4 | 2.14(1.51,3.02) | ***<0.001*** | 1.91(1.33,2.74) | ***0.001*** |
| Baseline CA19-9 level>2000 U/mL | 1.98(1.41,2.79) | ***<0.001*** | 1.50(1.04,2.17) | ***0.031*** |
| Tumor maximum cross-sectional rate change ratio <-0.1 | 1.61(1.13,2.29) | ***0.008*** | 1.49(1.04,2.13) | ***0.028*** |
| Emergence of new lesions | 1.96(1.38-2.78) | ***<0.001*** | 1.72(1.19,2.50) | ***0.004*** |

| **Table S3. Frequency distribution under different stratification** | | |
| --- | --- | --- |
| **Type** | **Score** | **n** |
| Binary classification | 8-9 | 31 |
|  | 10-13 | 124 |
| Three-group classification | 8-9 | 31 |
|  | 10-11 | 59 |
|  | 12-13 | 65 |

| **Table S4. Median OS for each group in three cohorts** | | | | |
| --- | --- | --- | --- | --- |
| **Prognosis status** | **cPD score** | **Median OS** | | |
|  |  | **Training Cohort** | **Validation Cohort 1** | **Validation Cohort 2** |
| CP | 8-9 | 6.27 | 5.43 | 8.60 |
| PP | 10-11 | 8.87 | 9.40 | 15.27 |
| GP | 12-13 | 12.20 | 13.70 | 18.93 |

| **Table S5. Baseline comparison between Keep and Alter groups** | | | | | | |
| --- | --- | --- | --- | --- | --- | --- |
| **Variable** | **Training Cohort** | | | **Validation Cohort 1** | | |
|  | **Keep(n=77)** | **Alter(n=55)** | ***p*** | **Keep(n=140)** | **Alter(n=35)** | ***p*** |
| ECOG PS at 1^st^ chemotherapy | 0.53 ± 0.50 | 0.56 ± 0.54 | 0.800 | 0.78 ± 0.45 | 0.89 ± 0.63 | 0.409 |
| ECOG PS at 1^st^ assessment | 0.84 ± 0.37 | 0.73 ± 0.59 | 0.104 | 0.89 ± 0.36 | 1.03 ± 0.51 | 0.069 |
| ECOG worsening | 24(31.2%) | 17(30.9%) | 0.975 | 24(17.1%) | 6(17.1%) | 1.000 |
| Charlson comorbidity index | 6.49 ± 2.26 | 7.02 ± 2.08 | 0.177 | 6.61 ± 2.16 | 7.31 ± 2.11 | 0.084 |

| **Table S6. Median OS for Each Group of Keep or Alteration of Treatment Regimens in the Training Cohort and Validation Cohort 1** | | | | | |
| --- | --- | --- | --- | --- | --- |
| **Cohort** | **Group** | **Median OS** | | ***p*** | **HR (95%CI)** |
|  |  | **Alter** | **Keep** |  |  |
| Training Cohort | CP | 7.17 | 6.23 | 0.9026 | 0.95(0.4-2.22) |
|  | PP | 7.82 | 11.15 | ***0.0061*** | ***0.44(0.24-0.8)*** |
|  | GP | 8.27 | 17.63 | ***<0.0001*** | ***0.24(0.13-0.43)*** |
| Validation Cohort 1 | CP | 6.25 | 7.00 | 0.6347 | 0.77(0.26-2.23) |
|  | PP | 7.8 | 9.57 | ***0.0198*** | ***0.5(0.27-0.9)*** |
|  | GP | 8.43 | 14.53 | ***0.0029*** | ***0.35(0.17-0.72)*** |
